# Evolution of sensitivity to warning cues from kin in plants with a structured population

**DOI:** 10.1002/ece3.11057

**Published:** 2024-02-21

**Authors:** Atsushi Yamauchi, Junji Takabayashi, Kaori Shiojiri, Richard Karban

**Affiliations:** ^1^ Center for Ecological Research Kyoto University Otsu Japan; ^2^ Department of Agriculture Ryukoku University Otsu Japan; ^3^ Department of Entomology and Nematology University of California Davis California USA

**Keywords:** HIPVs, induced anti‐herbivore resistance, kin competition, kin recognition

## Abstract

Plants exchange a variety of information intra‐ and interspecifically by using various mediating cues. For example, plant individuals that are injured by herbivores release volatile chemicals, which induce receiver plants to express anti‐herbivore resistance. Remarkably, some plant species were known to represent kin specificity in the response, where cues from a damaged individual induce a higher level of resistance in a kin receiver than in a non‐kin receiver. Such higher sensitivity to warning cues from kin could be advantageous via two mechanisms. If each herbivore tends to attack plants with a certain genotype, plants should be more sensitive to warning cues from kin that share genetic properties. In addition, if herbivores successively attack the neighboring plant with a high probability, and if related plants tend to grow in close proximity, plants may be more sensitive to warning cues from neighboring kin under the presence of a trade‐off between sensitivity to kin and non‐kin. In the present study, we constructed a mathematical model including those mechanisms to investigate the evolutionary process of the higher sensitivity to warning cues from kin than sensitivities to cues from non‐kin. According to the analysis of evolutionary dynamics, we revealed that both mechanisms could contribute, although higher sensitivity to cues from kin is more likely to evolve when the spatial range of competition is greater than the range of effective alarm cues. This result highlights the importance of the competition regime in the evolution of signaling among kin.

## Graphical abstract

Plant individuals that are injured by herbivores release volatile chemicals, which induce receiver plants to express anti‐herbivore resistance. Some plant species were known to represent kin specificity in the response, where the warning cues induce a higher level of resistance in a kin receiver than in a non‐kin receiver. Why does the receiver become more sensitive to the cues sent by kin? To answer this question, we theoretically investigated mechanisms that drive the evolution of higher sensitivity to warning cues from kin.

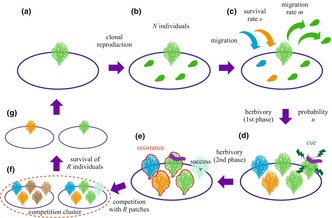

## 1 INTRODUCTION

It is widely recognized that plants exchange a variety of information intra‐ and interspecifically by using various types of mediating cues (Karban, 2021; Ninkovic et al., 2021). In particular, plant individuals that are injured by herbivores are known to release volatile chemicals called herbivore‐induced plant volatiles (HIPVs). HIPVs trigger diverse intra‐ and interspecific responses, for example, induction of anti‐herbivore resistance in other plant individuals in advance of herbivore attack (Heil & Karban, 2010; Karban, 2011, 2021; Karban et al., 2014). It is also known that HIPVs can be a signal that crosses trophic levels, for example, they attract predators that act as “bodyguards” of the plants (Godfray, 1995; Sabelis et al., 2007; Sabelis & de Jong, 1988; Takabayashi et al., 2006). These studies indicated that airborne plant chemicals are important communication cues for plants.

Plants are also known to recognize their kin in this communication process (Bilas et al., 2021; Karban, 2021). Some studies suggested that kin recognition is mediated by root exudates (Biedrzycki et al., 2010), causing plant individuals to modify their properties, including root growth (Dudley & File, 2007; File, Klironomos, et al., 2012; File, Murphy, & Dudley, 2012). In those cases, changes in individual properties following kin recognition are thought to reduce competition among relatives to maximize inclusive fitness (Dudley et al., 2013; Ehlers & Bilde, 2019). On the other hand, it is also known that HIPV communication among kin protects against future herbivory in plants. Cues from a damaged individual were reported to induce a higher level of anti‐herbivory resistance in a kin receiver than in a non‐kin receiver in sagebrush (*Artemisia tridentata*) (Karban, 2021; Karban et al., 2013; Karban & Shiojiri, 2009), lodgepole pine trees (*Pinus contorta*) (Hussain et al., 2019), and tall goldenrod (*Solidago altissima*) (Kalske et al., 2019; Shiojiri et al., 2021).

The evolution of warning cues has been discussed mainly with reference to animal behaviors, for example, alarm calls for detecting predators. The studies focused on various mechanisms involved in alarm recognition (Sherman, 1977; Smith, 1986), including kin selection (Tamachi, 1987). Animals are likely to give alarm signals before a predator attack, whereas plants release warning cues after suffering herbivory (some animals, e.g., aquatic animals, may also release the signals after injury by predators; Meuthen et al., 2014). The risk and cost of emitting signals/cues can differ between those two cases, for example, an alarm call before predation may attract the attention of the predator. In addition to this, the previous studies of alarm signals often assumed the absence of kin specificity, sharing the signals within a group including both relatives and non‐relatives (Sherman, 1977; Smith, 1986; Tamachi, 1987). However, the kin specificity that was reported in plant responses to HIPVs suggests that the evolution of kin recognition could make sense in warning signals/cues.

Communication generally comprises two types of players, that is, sender and receiver. In order for kin recognition to work in the communication system, the sender must send cues that specifically represent its genetic identity, while the receiver chooses specific behaviors or physiological changes in response to the specific cue. For the establishment of kin recognition, these two traits should evolve jointly. Penn and Frommen (2010) categorized mechanisms of kin recognition into familiarity‐dependent recognition and familiarity‐independent recognition, the latter of which included indirect familiarity, self‐inspection, and green‐beard genes. The green‐beard genes are genes representing genetic identities of individuals (Dawkins, 1976), which was originally postulated by Hamilton (1964b) in studies of the evolution of altruism. Among the mechanisms of kin recognition, green‐beard genes may be possible for organisms without intelligence like plants. Green‐beard traits have been reported in various organisms (Gardner & West, 2010; West & Gardner, 2010) but have not been detected in plants yet.

On the other hand, Karban (2021) pointed out the possibility that the release of informative cues may be unavoidable in plants, potentially leading to the evolution of communication as a byproduct. In such instances, the original function of HIPVs may not primarily entail the expression of kinship, despite containing certain information regarding the relatedness of the sender, which served as the basis for the evolution of kin recognition in receivers. An example of this phenomenon can be exemplified by the discernment observed among individual ants, wherein a pivotal determinant is the combination of hydrocarbons present on the cuticular surface of each individual. An ant perceives the status of another individual by juxtaposing the perceived label with an internal representation of its own colony's olfactory signature (Bos & d'Ettorre, 2012). The primary function of hydrocarbons resides in their role as a defensive waxy barrier that mitigates desiccation (Walsh et al., 2020), thereby indicating the partial preexistence of a cue prior to the evolution of recognition. Furthermore, it has been observed that the recognition of nestmates is contingent upon the context, whereby ants exhibit varying levels of aggression toward conspecifics under different circumstances (Sturgis & Gordon, 2012). This implies that the recipient's response can be behaviorally or evolutionarily adapted even to identical stimuli. The precise mechanism underlying kin recognition in plants remains elusive, although the evolution of a response toward the inevitable emission of HIPVs holds a certain degree of validity, rendering the evolution of green‐beard markers inconsequential. Based on these considerations, we should pay more attention to the behavior of the signal receiver than the sender. It should be noted that even if the green beard may evolve for plants, kin recognition can evolve only when it results in some advantage for the receiver.

With respect to the receiver strategy, a critical question arises for the evolution of kin recognition in warning communication between two plant individuals. Since the warning cues include important information about a dangerous emergency (e.g., the occurrence of herbivores), the receiver should respond to the cues irrespective of relatedness to the sender. Why does the receiver become less sensitive to the cues sent by non‐kin? To consider this question, we should recognize the importance of costs of sensitivity and/or response to the cues. In the absence of costs, sensitivities to cues of both kin and non‐kin should inflate simultaneously. Therefore, the cost for sensitivity and/or response to the cues is necessary for the evolution of different responses to the warning cues (Shiojiri et al., 2021).

In the presence of a cost of sensitivity, kin specificity of plant responses could be explained by differences in the level of emergency associated with warnings between kin and non‐kin senders. Shiojiri et al. (2021) reported that in tall goldenrods, the arthropod community on the plants was different among plant genotypes, which suggested that plants respond to volatiles from genetically close plants because they would have similar herbivore communities. This mechanism could result in an advantage of a higher sensitivity to warning cues from kin. Moreover, if relatives tend to co‐occur in close proximity, herbivores may successively attack the neighboring relatives with a high probability. With such a population structure, it might be better for individuals to be sensitive to the warning cues from kin. In either case, the selective force for sensitivity to warning cues may depend on the life history of the plant. For example, when a high rate of seed dispersal causes individuals to have more frequent encounters and interactions with non‐relatives than with relatives, they should be more sensitive to warning cues from non‐relatives. Thus, we should investigate evolutionary conditions favoring higher sensitivity to specific warning cues by associating relative sensitivity with the life history of the plant, introducing those two possible mechanisms.

In species that exhibit any level of sociality, individuals generally affect each other via multiple types of interactions. For instance, even if individuals are altruistic, they have to share various resources to live (e.g., space and food), resulting in competition for those. Recently, theoretical studies pointed out that the evolution of sociality was significantly influenced by the interplay of multiple types of interactions (Ito & Doebeli, 2019; Yamauchi et al., 2018). In particular, Yamauchi et al. (2018) showed that a difference in spatial scales of interactions can be a critical factor in the evolution of social interactions. Since plants may have specific ranges over which warning cues and competition operate, the evolution of warning cues could be influenced by a variation in those spatial ranges.

To understand the difference in sensitivities to HIPVs from kin and non‐kin, we constructed a conceptual mathematical model that includes differential herbivore specificity to different plant strains, the population structure of plants, and competition for space among plant individuals. The model assumes a trade‐off between sensitivities to HIPVs from kin and non‐kin. Based on a theoretical analysis of evolutionary dynamics of the sensitivities, we revealed that both of the two mechanisms were potentially effective for the evolution of higher sensitivity to warning cues from kin compared to cues of non‐kin, although a spatial scale of competition could be a critical factor. This trend may be partly analogous to the evolutionary conditions favoring cooperation in a structured population (Platt & Bever, 2009; Taylor, 1992) as discussed later. We also showed that multiple equilibria might be possible in the evolution of kin selection in plants depending on the relationship between sensitivity to cues and efficiency of anti‐herbivore resistance.

## 2 MATHEMATICAL MODEL

Under limited dispersal, kin selection drives the evolution of social interactions with relatives. These processes have been studied theoretically and fitness functions adapted for selection within metapopulations have been proposed. Parvinen and colleagues considered a metapopulation with population dynamics in subpopulations that are connected by migration, in which birth, death, and migration events were assumed to occur continuously (Parvinen, 2002; Parvinen et al., 2003; Parvinen & Metz, 2008). Lehmann et al. (2016) modeled transition processes between states of subpopulations in a heterogeneous environment by using a Markov chain. Those studies formulated the basic reproduction ratio of a mutant type as a proxy of invasive fitness. Those approaches can be general tools to investigate the evolution of social interactions. However, those approaches did not consider the cooccurrence of multiple social interactions at different spatial scales. We require a specific model involving multiple factors to study the evolution of responses to warning cues under competition with varying spatial scales.

We considered a plant population inhabiting an infinite number of discrete patches, with individuals sharing HIPVs within each patch. Note that the patch is defined as a spatial unit corresponding to the range of HIPVs in the present study. For simplicity, we assumed a plant species with clonal reproduction, in which each clonal strain releases a specific blend of HIPVs that can be a cue for kin recognition. In this study, we have employed a discrete‐event model, where events occur sequentially. Specifically, in the warning cue, the process inherently consists of two temporally distinct phases. Initially, a herbivore attacks a specific plant individual, triggering the infested plant to emit HIPVs. Subsequently, the herbivore infests the receiver plants that have already initiated anti‐herbivore resistance in response to the HIPVs. Hence, such a process can only be accurately described by a discrete‐event model.

**FIGURE 1:**
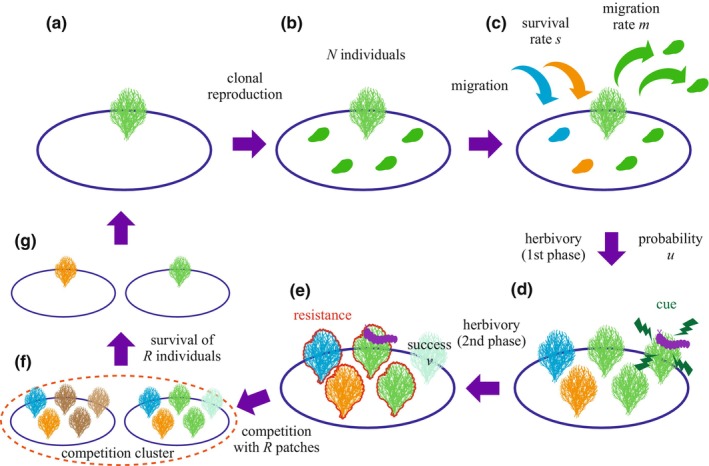
Schematic image of the plant lifecycle. (a) Initially, a patch is occupied by a single plant, (b) the plant reproduces clonally, (c) some individuals disperse, (d) a plant individual is infested by a herbivore and release warning cue, (e) other individuals express induced resistance in response to the cues, and are infested by the herbivore, (f) plant individuals among some patches (i.e., a competition cluster) compete for the patches, and (g) each patch is eventually occupied by a single‐plant individual in the next season.

A schematic image of the life cycle of the plant is illustrated in Figure 1. At the start of a season, a single individual occupies a patch. Each individual reproduces clonally, resulting in *N* individuals. A fraction *m* of the *N* individuals disperse from the natal patch over the entire population evenly and migrate to other patches with a probability *s*, that is, surviving dispersal. We assume that all patches include the same number of individuals after the dispersal event due to dispersing evenly. Each patch can harbor multiple unrelated immigrants due to the long dispersal range and the existence of a sufficiently large number of patches, where HIPVs are different among immigrants. Subsequently, each patch is attacked by an herbivore with a probability *u*, in which the herbivory progresses with two phases. In the first phase, the herbivore infests a plant individual, and the plant emits clone‐specific HIPVs. In this phase, the infested plant may or may not be exterminated despite the absence of resistance expression. In response to the HIPV warning cues, other members in the same patch express anti‐herbivore resistance. The sensitivity of receivers to HIPVs depends on the relatedness of the receivers in a patch, that is, either kin or non‐kin of the emitter. In the present study, we consider the sensitivity to the cues (HIPVs) as a trait that evolves. In the second phase of herbivory, the herbivore moves around within the patch and infests all other members evenly, except for the initially infested individual. In this phase, the amount of damage to each plant made by the herbivore was reduced by the resistance trait induced by HIPVs during the first phase.

After the herbivory, competition occurs among the individual plants that remain. The spatial scale of competition could be different from the scale of the patch that is a unit of sharing HIPVs. We do not consider that competition occurs between relatively adjacent patches (HIPV units), which could potentially lead to the exclusion of a strain occupying a patch (HIPV unit) following herbivory. We assume that competition occurs between *R* patches (*R* ≥ 1), by which *R* individuals eventually survive and become patch owners in the next generation. We consider that the migrants evenly disperse over a sufficiently large habitat. In this case, when some patches compete with the focal patch, those are unlikely to include individuals emigrating from the focal patch. Therefore, there is no relatedness among patches that compete.

In the evolution of sensitivity to cues, costs associated with detecting and responding to cues are critical factors. In the absence of costs, the sensitivity would increase as high as possible to achieve the maximum resistance level. Since this may be unrealistic, we introduced a cost of sensitivity to the HIPVs. It should be remarked that we could consider multiple types of costs. One type of cost is investment in the establishment of sensitivity, in which sensitivities to cues from kin and non‐kin can be independently determined by investing differentially in these two sensitivities. Another type of cost arises if the total resource pool is limited, which results in a trade‐off between sensitivities to cues from kin and non‐kin. In the present study, we adopt a trade‐off between sensitivities to cues from kin and non‐kin. Namely, specialization to kin reduces responsiveness to non‐kin, whereas ignorance of kin can improve responsiveness to non‐kin. Therefore, individuals are more sensitive to detecting cues emitted by kin, detect all signals equally, or are more sensitive to cues given off by non‐kin. We denote the individual allocation to sensitivities to cues from kin and non‐kin as *x* and 1 − *x*, respectively, where *x* is a strategy to evolve. In the present study, we assume that *x* can be smaller than 0.5, where plants are less sensitive to cues from kin than to cues from non‐kin. If the sensitivity to cues from kin should not be below those from non‐kin, *x* is bounded by 0.5, at which there is no sensitivity. Our analysis is also possible with a condition of 0.5 ≤ *x* ≤ 1, although we consider 0 ≤ *x* ≤ 1 to illustrate general evolutionary trends. When *x* = 1/2, the sensitivities to cues from kin and non‐kin are identical, implying the absence of bias in the sensitivity. It can be a standard of the trend in evolution of sensitivities.

**FIGURE 2:**
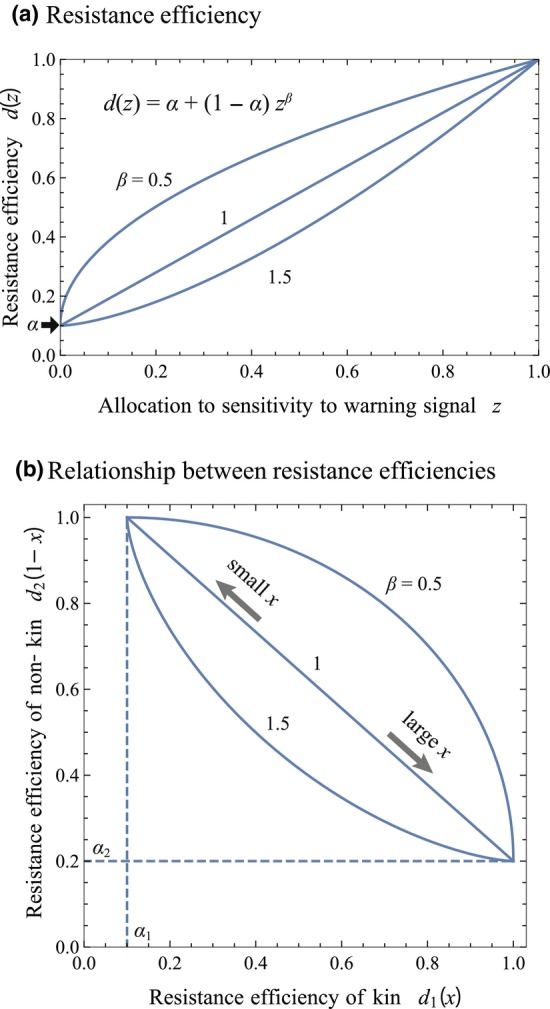
(a) Functional form of efficiency of induced resistance on the allocation to sensitivity to warning cues which is used in the present analysis. The function is assumed to pass through (0, *α*) and (1, 1) with various concavities, where *α* and *β* are plant survivorship without resistance and a curvature parameter, respectively. (b) Trade‐off between resistance efficiencies to herbivores that infest kin and non‐kin plants with varying sensitivity to cues from kin plants, *x*. The trade‐off is concave, linear, and convex with *β* > 1, *β* = 1, and *β* < 1, respectively.

We assume that the initially infested individual remains after the first phase of herbivory, and can avoid the second phase of herbivory, eventually surviving infestation with a probability *v* (< 1) (see Figure 1). On the other hand, the success of plants infested in the second phase of herbivory depends on their level of induced anti‐herbivore resistance. Thus, we should also consider a relationship between the sensitivity and the level of induced anti‐herbivore resistance. It could be considered that a larger allocation of resources to sensitivity will result in expression of greater resistance, although the relationship may not be linear. For example, it might be possible that the resistance level increases rapidly due to a greater allocation of resources. Accordingly, we represent a resistance efficiency, *d*(*z*) = *α* + (1 − *α*) *z* ^*β*^ (<1), as a function of the allocation to sensitivity to cues, *z* (*z* ∊ *x*, 1 − *x*), which is the survivorship of a plant that allocates to the sensitivity to specific cues with allocation level *z*. The function increases with z, which passes through (0, *α*) and (1, 1), being the maximum *d*(1) = 1 at the allocation level *z* = 1. In this function, *α* represents a basal resistance efficiency in the absence of sensitivity (*z* = 0), and *β* determines the curvature of the function. Examples of *d*(*z*) are illustrated in Figure 2a.

Importantly, the damage level of plants infested in the second phase may depend on relatedness to the plant infested in the first phase. If the herbivores have some specificity to plant genotypes or strains, the herbivores that initially attack a certain plant tend to preferentially damage kin of this plant in the second phase of herbivory. Here, we consider the process of herbivory in more detail, incorporating the specificity of herbivores. We assume that all clonal strains of plants have specific herbivores and that herbivores randomly visit plant patches and initiate the first phase of herbivory when finding a plant that matches their specifications. The frequencies of these matches in the herbivore population are considered to follow a uniform distribution, which justifies a random encounter of a plant with specific herbivores. In the second phase of herbivory, the herbivore attacks other plant individuals in the patch, where the damage to non‐specific plants may be less severe. Thus, kin and non‐kin plants of the initially infested plant are accompanied by the resistance efficiency functions

(1a) $$d_{1}\left(z\right)=\alpha_{1}+\left(1-\alpha_{1}\right)\;z^{\beta },\text{and}$$

(1b) $$d_{2}\left(z\right)=\alpha_{2}+\left(1-\alpha_{2}\right)\;z^{\beta },$$

respectively, with different basal resistance efficiencies *α*_1_ and *α*_2_. If the initially infested plant is an immigrant in the patch, all secondary infested plants experience the resistance efficiency *d*_2_(*z*) due to the genetic dissimilarity with the immigrant. When the initially infested plant is natal in the patch, its kin also exists there, representing the resistance efficiency *d*_1_(*z*). If kin of the initially infested plant tend to suffer more severe damage in the second phase of herbivory, it satisfies *α*_1_ < *α*_2_.

It should be noted that we consider a linear negative relationship between allocations to sensitivities to signals from kin and non‐kin, that is, *x* and 1 − *x*, although it can result in a non‐linear trade‐off between resistance efficiencies to herbivores that have infested kin and non‐kin plants. Figure 2b illustrates the relationship between *d*_1_(*x*) and *d*_2_(1 − *x*), which indicates that the curvature of the trade‐off between resistance efficiencies depends on *β*‐value. Therefore, the present model substantially involves various trade‐off relationships between the effects of sensitivities to cues from kin and non‐kin plants.

Based on the above assumption, we formulate probabilities that individuals of each clonal strain become patch owners in the next generation, which can be regarded as success of the clonal strains. The formulation would vary depending on migration processes. The number of immigrants in a patch is represented by *smN*. When *smN* is greater than 1, we assume that all patches always involve *smN* immigrants. On the other hand, if *smN* is smaller than 1, we consider that patches include 0 and 1 immigrant with probability 1 − *smN* and *smN*, respectively, where an expected immigrant number is 0 × (1 − *smN*) + 1 × *smN* = *smN*. This operation is adopted to avoid artifacts in the formulation (see below). In the following, we explain the formulation, distinguishing those two cases.

### 2.1 Case 1: Each patch includes more than or equal to 1 immigrant on average ( *smN* ≥ 1)

When *smN* is greater than 1, it is assumed that all patches always involve *smN* immigrants. In this case, the expected numbers of individuals in a patch that survive after the second phase of herbivory are described in Table 1. Table 1 includes four outcomes depending on the strain that is infested in the first phase of herbivory (categorized by *i* = 1, 2, 3, and 4). *F*_*i*_(*x*), *G*_*i*_(*x*), and *H*_*i*_(*x*) represent the number of surviving individuals of the natal strain of the patch, a focal immigrant, and other immigrants, respectively. It should be noted that other immigrants can include clones of multiple strains, among which there is no kinship.

**TABLE 1:** Expected number of individuals in a patch after herbivory under *smN* ≥ 1.

	Probability	Expected number of surviving individuals in a patch after herbivory		
		Natal individuals (*x*_1_)	A focal immigrant (*x*_2_)	Other immigrants (*x*_3_)
First herbivory				
A natal individual	$p_{1}=u\frac{\left(1-m\right)N}{\left[1‐\left(1‐s\right)m\right]N}$	*F*_1_(*x*_1_) = *v* + *d*_1_(*x*_1_)[(1 − *m*)*N* − 1]	*G*_1_(*x*_2_) = *d*_2_(1 – *x*_2_)	*H*_1_(*x*_3_) = *d*_2_(1 − *x*_3_)(*smN* − 1)
A focal immigrant	$p_{2}=u\frac{1}{\left[1‐\left(1‐s\right)m\right]N}$	*F*_2_(*x*_1_) = *d*_2_(1 − *x*_1_)(1 − *m*)*N*	*G*_2_(*x*_2_) = *v*	*H*_2_(*x*_3_) = *d*_2_(1 − *x*_3_)(*smN* − 1)
Another immigrant	$p_{3}=u\frac{\mathrm{smN}-1}{\left[1‐\left(1‐s\right)m\right]N}$	*F*_3_(*x*_1_) = *d*_2_(1 − *x*_1_)(1 − *m*)*N*	*G*_3_(*x*_2_) = *d*_2_(1 − *x*_2_)	*H*_3_(*x*_3_) = *v* + *d*_2_(1 − *x*_3_)(*smN* − 2)
No herbivory	1 − *u*	*F*_4_(*x*_1_) = (1 − *m*)*N*	*G*_4_(*x*_2_) = 1	*H*_4_(*x*_3_) = *smN* − 1

To analyze the evolutionary dynamics of sensitivity, we examine the invasibility of a rare mutant strain allocating *x* to sensitivity to cues from kin, in the population comprising resident strain with $\bar{x}$. Based on Table 1, we denote the total number of survivors as a summation of *F*_*i*_(*x*), *G*_*i*_(*x*), and *H*_*i*_(*x*) with various combinations of resident and mutant individuals as

(2a) $$K_{i}=F_{i}\left(\bar{x}\right)+G_{i}\left(\bar{x}\right)+H_{i}\left(\bar{x}\right),$$

(2b) $${\hat{K}}_{i}=F_{i}\left(x\right)+G_{i}\left(\bar{x}\right)+H_{i}\left(\bar{x}\right),$$

(2c) $${\overset{ˇ}{K}}_{i}=F_{i}\left(\bar{x}\right)+G_{i}\left(x\right)+H_{i}\left(\bar{x}\right),$$

which represent survivors in a patch with resident individuals only, an original patch of the mutant, and an original patch of the resident strain with a single mutant immigrant, respectively.

In the subsequent competition, *R* patches form a cluster for competition, which represents a spatial scale of competition (see (f) in Figure 1). We assumed *R* to be a natural number. We concentrate on the anticipated quantity of patches that an individual mutant, originating at the commencement of the season, ultimately acquires by the season's end. This quantity can be deemed as the mutant's fitness. The fitness is determined by considering the probability of a mutant being randomly chosen in the competition. It is noteworthy that the number of individuals in the competition cluster is contingent upon the arrangement of R patches undergoing diverse herbivory processes. The probability of such arrangement follows a multinomial distribution with respect to the patch types. Consequently, we can express the mutant's fitness using the multinomial distribution as
(3)

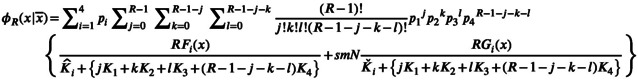




The first and second terms within curly brackets represent an expected number of mutants selected in the competition, for a mutant‐initiating patch and resident‐initiating patches with a single mutant immigrant, respectively. Since *smN* individuals can successfully emigrate from a mutant patch, it is multiplied to the second term. In those terms, a denominator is the total number of individuals in the cluster of *R* patches, including the focal patch, and patches of residents with specific modes of the first phase of herbivory with a combination of 1, *j*, *k*, *l*, and *R* − 1 − *j* − *k* − *l* (the total is *R*). In the cluster formation, the mutants are included only in the focal patch, whereas the other *R* − 1 patches comprise the resident strain only due to the rarity of the mutant strain. The expected number of selected mutants is averaged for a combination of *j*, *k*, *l*, and *R* − 1 − *j* − *k* − *l* with the multinomial distribution. Finally, this number is averaged for modes of the first phase of herbivory in the focal patch with probability *p*_*i*_ (see Table 1).

### 2.2 Case 2: Each patch includes fewer than 1 immigrant on average ( *smN* < 1)

When *smN* is smaller than 1, we cannot use the same approach as in the previous case because the number of surviving individuals becomes negative for some values in Table 1. Therefore, we formulate a fitness under *smN* < 1 by combining cases in the absence and the presence of a single immigrant in a patch. The expected numbers of surviving individuals after herbivory are shown in Table 2 for five cases concerning the presence or absence of an immigrant, and nature of the strain infested in the first phase of herbivory. Consider a rare mutant strain with sensitivity *x* in the population comprising resident strain with $\bar{x}$. The total number of survivors in the five cases with various combinations of resident and mutant individuals are

(4a) $$K_{i}=F_{i}\left(\bar{x}\right)+G_{i}\left(\bar{x}\right),$$

(4b) $${\hat{K}}_{i}=F_{i}\left(x\right)+G_{i}\left(\bar{x}\right),$$

(4c) $${\overset{ˇ}{K}}_{i}=F_{i}\left(\bar{x}\right)+G_{i}\left(x\right),$$

which are survivors in a patch with resident individuals only, an original patch of the mutant, and an original patch of the resident strain with a single mutant immigrant, respectively.

**TABLE 2:** Expected number of individuals in a patch after herbivory under *smN* < 1.

	Probability	Expected number of surviving individuals in a patch after herbivory	
		Natal individuals (*x*_1_)	A focal immigrant (*x*_2_)
Presence of an immigrant			
First herbivory			
A natal individual	$p_{1}=\text{smNu}\frac{\left(1-m\right)N}{\left(1-m\right)N+1}$	*F*_1_(*x*_1_) = *v* + *d*_1_(*x*_1_)[(1 − *m*)*N* − 1]	*G*_1_(*x*_2_) = *d*_2_(1 − *x*_2_)
A focal immigrant	$p_{2}=\text{smNu}\frac{1}{\left(1-m\right)N+1}$	*F*_2_(*x*_1_) = *d*_2_(1 − *x*_1_)(1 − *m*)*N*	*G*_2_(*x*_2_) = *v*
No herbivory	$p_{3}=\mathrm{smN}\left(1-u\right)$	*F*_3_(*x*_1_) = (1 − *m*)*N*	*G*_3_(*x*_2_) = 1
Absence of immigrant			
First herbivory			
A natal individual	$p_{4}=\left(1-\mathrm{smN}\right)u$	*F*_4_(*x*_1_) = *v* + *d*_1_(*x*_1_)[(1 − *m*)*N* − 1]	*G*_4_(*x*_2_) = 0
No herbivory	$p_{5}=\left(1-\mathrm{smN}\right)\left(1-u\right)$	*F*_5_(*x*_1_) = (1 − *m*)*N*	*G*_5_(*x*_2_) = 0

Similar to the case of *smN* ≥ 1, we can calculate an expected number of patches that a single mutant at the start of the season eventually occupies at the end of the season as
(5)

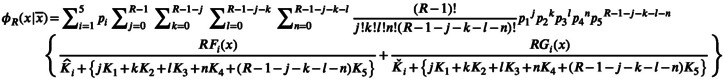




We assumed *R* to be a natural number. The structure of Equation (5) is basically identical to that of Equation (3), although there is a difference in the treatment of *smN*. In the case of *smN* ≥ 1, *smN* represents a number of surviving emigrants from a mutant patch; therefore, *smN* is multiplied by the second term within curly brackets of Equation (3), which is a probability for selecting a mutant individual in the competition. On the other hand, in the case of *smN* < 1, *smN* is considered as a possibility of existence of a single immigrant in the patch; therefore, *smN* is involved in the probability of occurrence of each case *p*_*i*_ in Equation (5) (see Table 2).

### 2.3 Generalizing fitness formulation for real numbers of *R*

In both Equations (3) and (5), we assume that the number of patches forming a cluster for competition, *R*, is a natural number. Based on Equations (3) and (5), we can calculate the expected number of patches that a single mutant at the start of the season eventually obtains at the end of the season, with a real number of *R* (*R* ≥ 1). It can be considered that when *R* is a real number, the size of the competition cluster becomes Floor[*R*] and Floor[*R* + 1] with probabilities Floor[*R* + 1] − *R* and *R* − Floor[*R*], respectively, where Floor[*z*] is a floor function giving the greatest integer less than or equal to *z*. Thus, the expectation is

(6) $$\psi \left(x\;|\bar{x}\right)=\left(\text{Floor}\left[R+1\right]-R\right)\;\phi_{\text{Floor}\left[R\right]}\left(x|\bar{x}\right)+\left(R-\text{Floor}\left[R\right]\right)\;\phi_{\text{Floor}\left[R+1\right]}\left(x|\bar{x}\right),$$

by using Equation (3) or (5) depending on the value of *smN*. Equation (6) can be regarded as a total success of the mutant strain, which integrates individual successes in various cases of interactions among kin over the communication and competition stages. We investigate the evolution of the sensitivity *x* to warning cues from kin by analyzing Equation (6) based on the adaptive dynamics theory (Geritz et al., 1998).

It should be remarked that Equation (6) generally became 1 under *x* = $\bar{x}$ with either Equations (3) or (5), which indicated that the resident fitness was always 1, suggesting that a single resident individual can obtain a single patch in the resident population. In Tables 1 and 2, some values can be negative with (1 − *m*)*N* < 1, implying that the formulation was not valid if almost all individuals left the natal patch. To avoid this artifact, we focused on the case with (1 − *m*)*N* > 1, that is, *m* < 1–1/*N*.

The total success of the mutant strain Equation (6) was too complex to analyze, although first‐order and second‐order derivatives can be obtained by using Mathematica 12 (Wolfram Research, Inc.). We could not process those derivatives analytically due to the complexities, but we could analyze them numerically. We searched evolutionary equilibria by numerically analyzing the zero selection gradient (i.e., ∂*ψ*($\bar{x}$|$\bar{x}$)/∂*x* = 0), and examined those convergent and evolutionary stabilities by numerically analyzing the second order of derivatives at the equilibria (i.e., ∂(∂*ψ*($\bar{x}$|$\bar{x}$)/∂*x*)/∂$\bar{x}$ < 0 and ∂^2^ *ψ*($\bar{x}$|$\bar{x}$)/∂*x* ^2^ < 0, respectively). In addition, we also analyzed evolutionary stabilities for boundary conditions, *x* = 0 and *x* = 1, by checking the signs of selection gradients at the boundaries. In the calculation, the probability of appearance of herbivore, *u*, becomes just a coefficient after differentiation, therefore, it does not influence the equilibrium state substantially.

## 3 RESULTS

**FIGURE 3:**
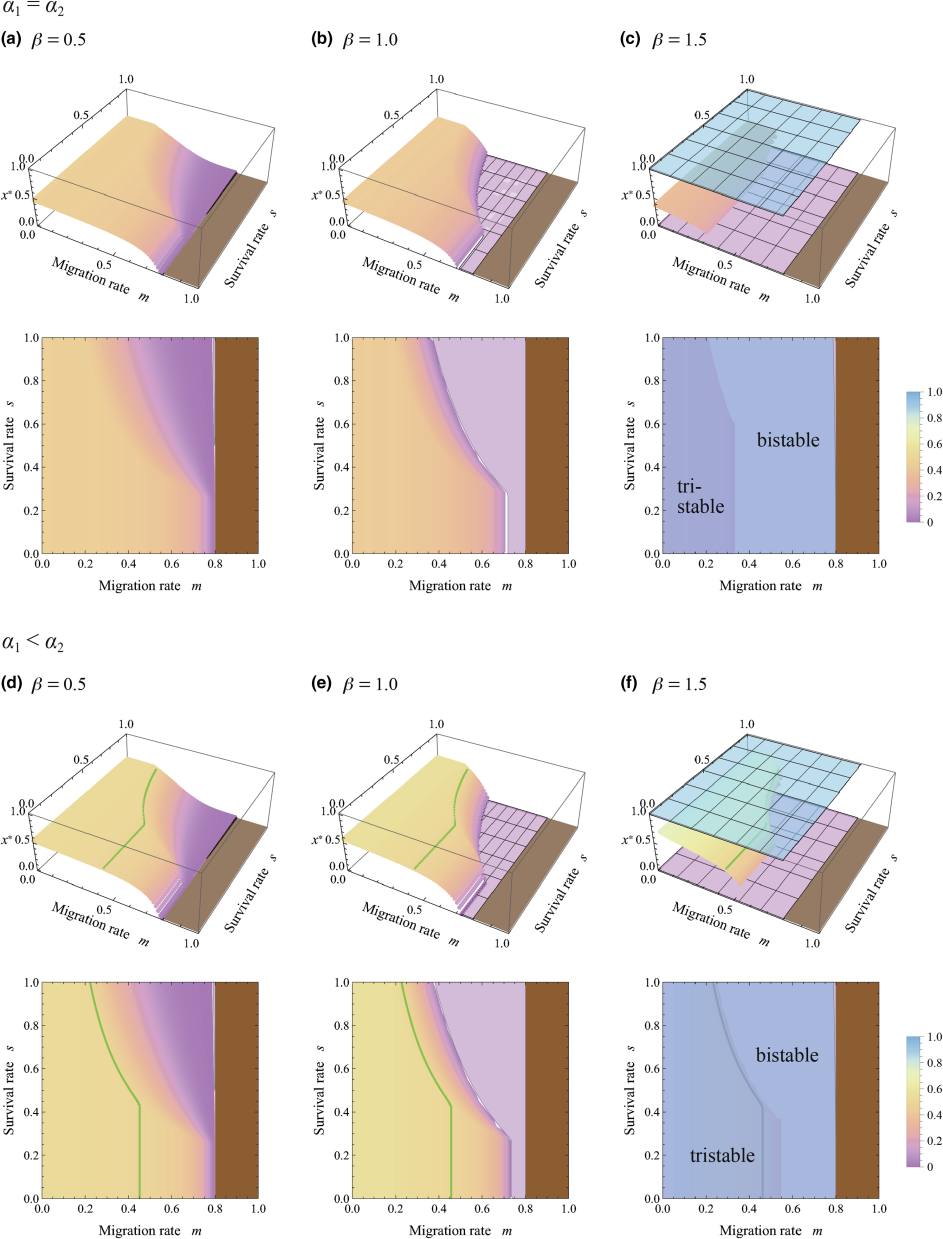
Evolutionarily and convergently stable allocation to sensitivity to warning cues from kin, x*, with varying migration rates *m* and survival rates *s*. Colors represent the level of x*, which correspond with the color chart on the right side. In each panel, an identical result is plotted in both 3D and contour plots. A brown region is excluded from analysis due to less than 1 individual remaining in the natal patch, that is, (1 − *m*)*N* < 1. A green curve represents a contour of *x* = 0.5. Parameters are *R* = 1, *N* = 5, *v* = 0.2, *α*_1_ = 0.2, and *α*_2_ = 0.2 in (a–c), and *α*_2_ = 0.4 in (d–f). (Although *u* = 1, this value does not affect the result.)

First, we investigated stable equilibria of allocation to sensitivity to cues from kin, x*, by assuming that the spatial scale is identical between communication involving HIPVs and competition, that is, *R* = 1. Figure 3a–c plotted results in the absence of herbivore specificity to plant strains (*α*_1_ = *α*_2_); in this scenario, the success of a secondarily infested plant does not depend on its kinship to the initially infested plant. These figures showed that with *R* = 1, interior equilibria (0 < x* < 1) cannot exceed 0.5, implying that higher sensitivity to warning cues from kin was unlikely to evolve. When the resistance efficiency in response to allocation to specific cues was a convex function (*β* > 1), perfect sensitivity to the warning cues from kin (x* = 1) is possible, although the moderately higher sensitivity to warning cues from kin (0.5 < x* < 1) is not possible. This result indicated that higher sensitivity to the warning cues from kin was difficult to evolve even in a highly structured population that was accompanied by aggregation of kin with low migration and low survivorship (i.e., small *m* and *s*).

In Figure 3d–f, we analyzed the model including herbivore specificity to particular plant strains (*α*_1_ < *α*_2_), where the secondary infested plants with kinship to the initially infested plant suffered more severe damage from the herbivores. According to the figures, the solutions of sensitivity to kin become slightly larger than those in Figure 3a–c, such that higher sensitivity to warning cues from kin can evolve with low migration and low survivorship (i.e., small *m* and *s*). Despite this promotional effect, the value of sensitivity tended to stay near x* = 0.5, implying that the sensitivity was weak. This result suggested that herbivore specificity to plant strains can promote the evolution of higher sensitivity to warning cues from kin, although the effect may be limited.

**FIGURE 4:**
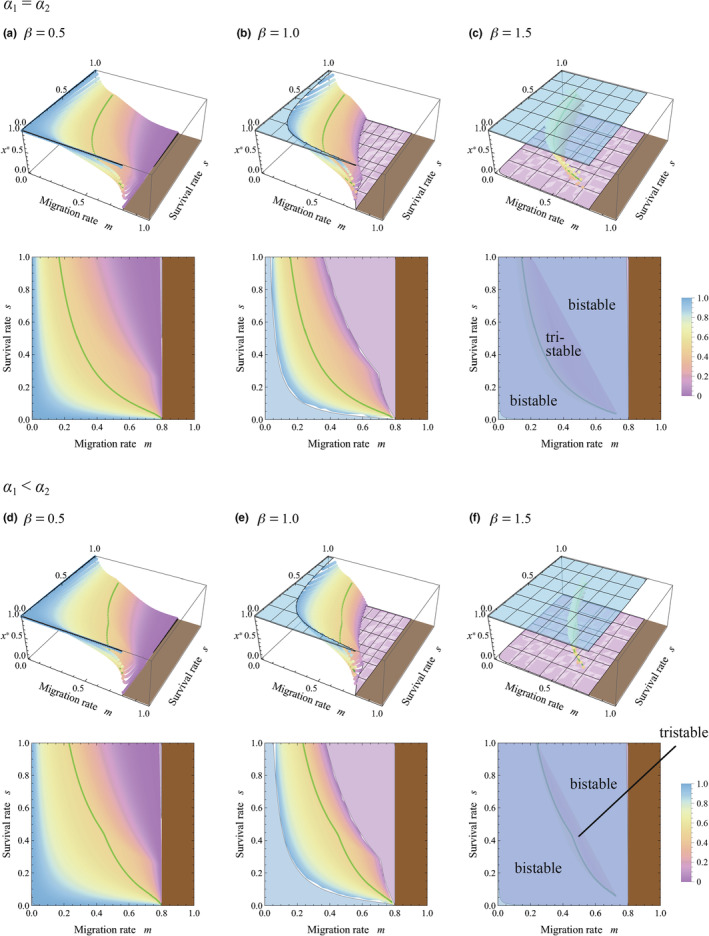
Evolutionarily and convergently stable allocation to sensitivity to warning cues from kin, x*, with varying migration rates *m* and survival rates *s*. The conditions and parameters are identical with those of Figure 3, except that now *R* = 1.1. (Although *u* = 1, the value does not affect the result.)

Next, we analyzed the model under the assumption that the spatial scale of competition is larger than the spatial scale of communication involving HIPVs, that is, *R* > 1. Figure 4 plotted stable equilibria of allocation to sensitivity to cues from kin, x*, under identical conditions and parameters to Figure 3, except for *R* = 1.1. Figure 4a–c showed that even in the absence of herbivore specificity to plant strains (*α*_1_ = *α*_2_), the higher sensitivity to warning cues from kin can evolve in the highly structured population with low migration and low survivorship (i.e., small *m* and *s*). Furthermore, Figure 4d–f indicated that the herbivore specificity to plant strains (*α*_1_ < *α*_2_) promoted the evolution of higher sensitivity to warning cues from kin. These results suggested that both population structure and herbivore specificity to plant strain could potentially promote the evolution of higher sensitivity to warning cues from kin, although those were effective only when the competition occurred over a larger spatial scale than HIPV sharing. It should be noted that the difference between two spatial scales need not be significant for this trend, and a slight difference could sufficiently enable the evolution of higher sensitivity to cues from kin (i.e., *R* = 1.1).

**FIGURE 5:**
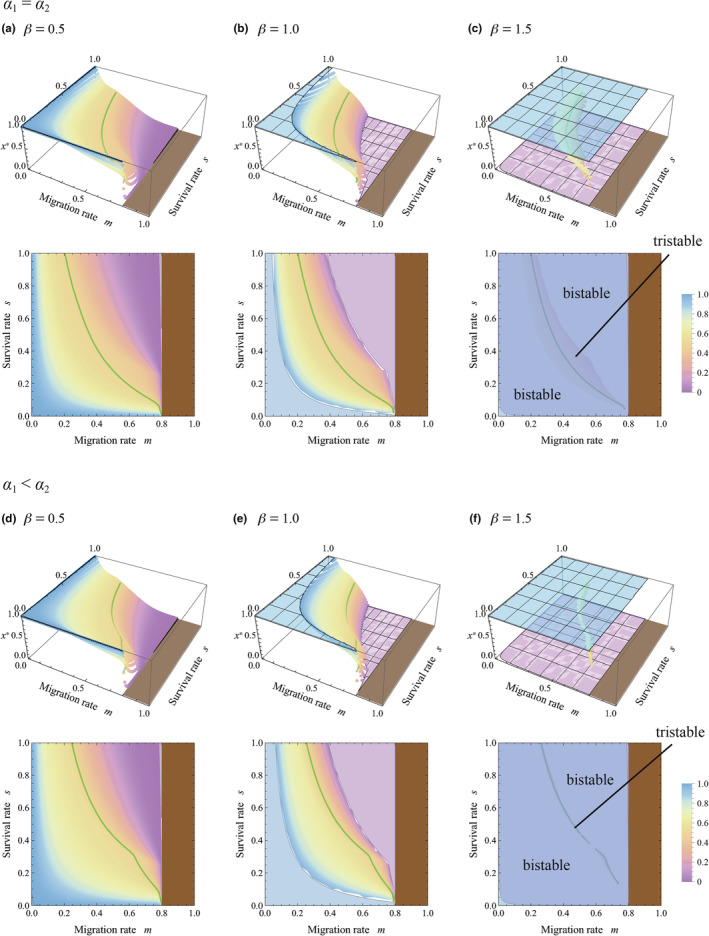
Convergently and evolutionarily stable allocation to sensitivity to warning cues from kin, x*, with varying migration rate *m* and survival rate *s*. Conditions and parameters are as Figure 4, except for *v* = 0.05.

**FIGURE 6:**
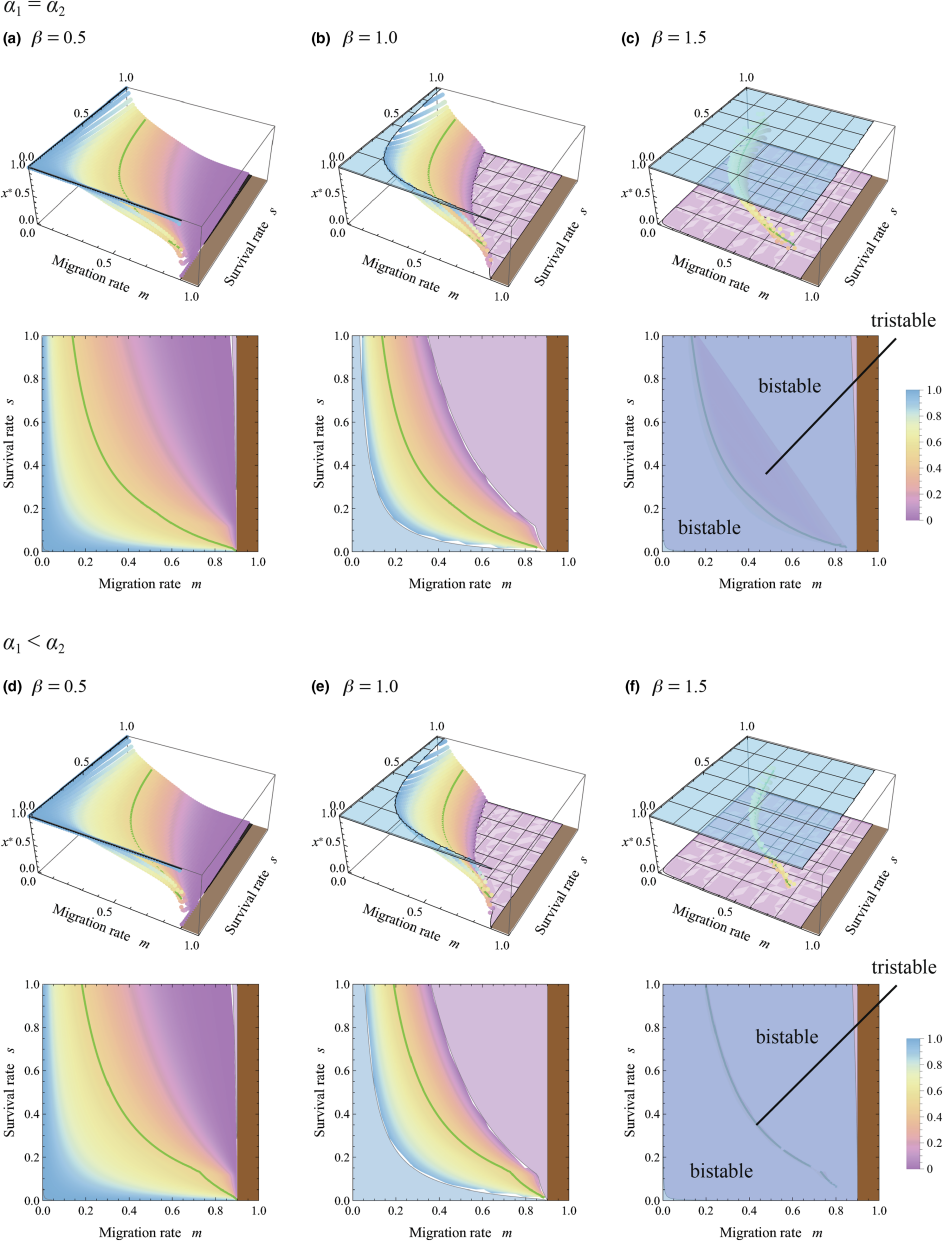
Convergently and evolutionarily stable allocation to sensitivity to warning cue from kin, x*, with varying migration rate *m* and survival rate *s*. Conditions and parameters are as Figure 4, except for *N* = 10.

We also checked the effects of other parameters on the sensitivity to cues from kin. We examined the effects of the success of the initially infested plant in the first phase of herbivory in Figure 5 under the identical conditions and parameters to Figure 4, except for the success of the initially infested individual, *v* (i.e., *v* = 0.2 in Figure 4 and *v* = 0.05 in Figure 5). The figure showed that when the initially infested plant suffered more severe damage from herbivores (i.e., *v* = 0.05 in Figure 5), the parameter region with x* > 0.5 became wider, implying that the evolution of higher sensitivity to warning cues from kin was promoted. We also examine the effects of patch size for HIPV sharing in Figure 6, in which the conditions and parameters are identical to Figure 4, except for *N* (i.e., *N* = 5 in Figure 4 and *N* = 10 in Figure 6). In comparing those figures, it is suggested that the increasing number of individuals in the patch could suppress the evolution of higher sensitivity to warning cues from kin to some degree (it is relatively clear in cases with *α*_1_ < *α*_2_ as Figure 6d–f).

In all cases of Figures 3, 4, 5, 6, multi‐stability occurs with *β* > 1, in which the equilibrium is bistability and tristability depending on the migration and survival rates. This suggests that when the resistance efficiency is a convex function of the allocation to sensitivity to warning cues (see Figure 2), the evolutionary consequence relies on the initial condition. It is remarkable that both x* = 0 and x* = 1 can be stable equilibrium in the wide range of the parameters, where sensitivity specializes toward cues from either kin or non‐kin conditionally. Those trends can result from the ineffectiveness of the intermediate level of sensitivity under the convex function of resistance efficiency, where generalists tend to suffer a disadvantage.

It should be noted that a high migration rate (large *m*) reduces kinship within a group. Figures 3, 4, 5, 6 demonstrate that when kin interaction is unlikely to occur under intense migration, plants typically maximize sensitivity to warning cues from non‐kin while minimizing that from kin. This is reasonable because warning cues originate solely from non‐kin in such cases.

## 4 DISCUSSION

The study of kin recognition has been explored within the framework of the Philip Sydney game (Johnstone & Grafen, 1992; Maynard Smith, 1991). In this game, the sender possesses two distinct states, namely “health” and “needy,” and selects corresponding behaviors, such as “signal” and “quiet,” for each state (Bergstrom & Lachmann, 1997). Based on the signal and the sender's kinship, the receiver determines whether to assist the sender or not. It is important to emphasize the notable differences between the Philip Sydney game and our system. First, our model incorporates sender states of “with infestation” and “without infestation,” with the sender being compelled to exhibit specific behaviors: “warning” and “no warning” for the respective states. Furthermore, the receivers engage in defensive expression against herbivory to enhance their individual fitness upon receiving the signal, which does not directly provide assistance to the sender. Importantly, the concept of inclusive fitness does not significantly impact the receiver during the direct sender–receiver interaction. However, in the competition phase, the survival of the receiver can affect the success of the sender, thereby necessitating the consideration of inclusive fitness. The cumulative effects of these dynamics are comprehensively evaluated through the formulation of the strain success metric.

The present analysis suggested that evolution of the higher sensitivity to cues from kin can be potentially promoted if the population was highly structured with low migration and low survivorship (i.e., small *m* and *s*), and if the secondarily infested plants with kinship to the initially infested plant suffered more severe damage from herbivores (*α*_1_ < *α*_2_). Remarkably, those promotional effects were effective only when the spatial scale of competition was larger than the scale of signaling, *R* > 1 (see Figure 4). In the sagebrush system that shows specific response to HIPVs from kin, the effective distances for plant–plant communication were ca. 50–60 cm (Karban et al., 2006). Since this species grows to a diameter of >1 m on average, the spatial scale of competition could be larger than the scale of signaling in this species, satisfying the condition for the evolution of higher sensitivity to warning cues from kin.

The effect of the difference in spatial scales for competition and communication leading to induced resistance may be partially analogous to the condition required for the evolution of cooperation with limited dispersal due to spatial structure. Intuitively, the limited dispersal increases interactions among kin, which may promote evolution of cooperation through kin selection. In reality, however, competition among kin cancels the effect of cooperation and prevents cooperation from evolving (Platt & Bever, 2009; Taylor, 1992). Similarly, a benefit of responding to warning cues from kin may be canceled by kin competition when the competition cluster is small. The presence of cues from kin implies the possible existence of kin within the same patch (even if the sender of the cue dies). Thus, the warning cues include information simultaneously of two kinds: the presence of herbivores and the presence of kin within the patch. In this case, induced resistance increases the success of the focal individual, which could, in turn, reduce the success of its kin via intensifying kin competition. This canceled the advantage of responding to the warning cues from kin.

According to the analysis, plants can be sensitive to warning cues from kin (x* > 0.5) or from non‐kin (x* < 0.5) under a wide range of conditions, where the sensitivity was unlikely to be neutral (see Figures 3 and 4). The relatedness‐dependent response to HIPVs (x* > 0.5) has been reported in sagebrush (Karban, 2021; Karban et al., 2013; Karban & Shiojiri, 2009), lodgepole pine tree (Hussain et al., 2019), and tall goldenrod (Kalske et al., 2019; Shiojiri et al., 2021), although the phenomena might not be general in other plant species. We should consider why the relatedness‐dependent response may not be general. According to the analysis, if the spatial scale of competition was equal to that of HIPV sharing (*R* = 1), the sensitivity is similar between cues from kin and non‐kin (x* ≈ 0.5); this situation is found in a wide range of combinations of migration and survival rates typically found when resistance efficiency, *d*(*z*), is a concave or linear function (plateaus in Figure 3a,b,d,f). If the scale of competition is larger than the scale of signaling (*R* > 1), higher sensitivity to warning cues from kin would evolve. Even in this case, when the resistance efficiency function is strongly concave, the difference in sensitivities to cues from kin and non‐kin was relatively small within a wide parameter set (x* ≈ 0.5 in Figure 4a,d). In addition, severe damage to the initially infested plant (small *v*) promoted high sensitivity to cues from kin, which was also associated with an expansion of the parameter region with relatively low sensitivity, x* ≈ 0.5 (compare Figures 4 and 5). These factors may contribute to maintaining the specificity of plants for warning cues at a relatively low level.

The present study also showed that the evolution of higher sensitivity for cues from kin was suppressed when damage by herbivores to the initially infested individual was small (large relative success, *v*; see Figures 4 and 5), and when patch size for HIPV sharing was large (large *N*; see Figures 4 and 6). We can also explain those trends as effects of kin competition. When the initially infested individual belongs to a natal strain of the patch, its higher survivorship results in the existence of more kin in the patch. Thus, a kin receiver may suppress its response to cues from damaged kin to relax kin competition, improving the success of the clonal strain. If the patch size for HIPV sharing was small, each individual makes up a relatively large fraction of the population. In such a case, damage to an individual in the first phase of herbivory results in a significant reduction of relative density in the population, which weakens competition. In particular, if the herbivore infests an individual with the natal strain of the patch, it can notably reduce the kin competition. In the large patch, in turn, this effect becomes weak, where the kin competition tends to suppress the evolution of higher sensitivity for cues from kin.

It was reported that in tall goldenrod, receiver plants from populations with ambient herbivory generally induced resistance in response to cues from both damaged kin and non‐kin equally, whereas plants from populations without herbivores only responded to cues from kin (Kalske et al., 2019). This trend could be explained by results of the analysis presented here. We revealed that when the damage to secondarily infested plants did not depend on their kinship to the initially infested plant (*α*_1_ = *α*_2_), evolution of higher sensitivity to cues from kin was suppressed to some degree (see Figure 4). It might be possible that the high density of herbivores decreases their specificity to plant genotypes, due to severe competition for food resources. If it equalized the damage to kin and non‐kin plants in the second phase of herbivory, it may suppress the evolution of higher sensitivity to cues from kin, which could explain the observed trend.

In the model presented, we assumed that the plants reproduced clonally only, which does not fit many plant species. Sexual reproduction could reduce relatedness among siblings. In the non‐natal patches, emigrants never interact with kin, and relatedness among kin would not alter the selection pressure for higher sensitivity to cues from kin. In the natal patch, relatedness does not affect the significance of information in the HIPVs from kin, that is, a cue suggesting high probability of forthcoming herbivory. Thus, the siblings would be sensitive to cues from kin regardless of the relatedness as far as the cues from kin are discernible. Simultaneously, low relatedness moderates the kin competition in the natal patch, which could promote the evolution of higher sensitivity to cues from kin. Therefore, the decreasing relatedness in sexual reproduction may promote the evolution of higher sensitivity to cues from kin, which might be somewhat paradoxical.

The present analysis indicated that multi‐stability of equilibria was possible when the resistance efficiency, *d*(*z*), was a convex function of sensitivity to cues (Figures 3c,f, 4c,f, 5c,f and 6c,f). In those cases, sensitivity to warning cues varied depending on the initial conditions, suggesting the possibility of inter‐population variation in sensitivity. The phenomenon is interesting, although it might seem unrealistic. The convex shape of *d*(*z*) implied that a small investment in sensitivity could not notably improve resistance efficiency (see Figure 2a). If sensitivity is determined by a number of receptors sensing the specific blend of HIPVs, and if the activation of only a few receptors could trigger the chemical cascade of plant response to some degree, the shape of *d*(*z*) may not be convex, and the multi‐stability is unlikely to occur. This might be a possible situation, although further empirical study is necessary to answer this issue.

In this study, we investigated the evolution of the signaling system between kin, which was partly analogous with kin selection in the evolution of altruism. We formulated the total success of a clonal strain that compared the performances of individuals that responded differently to kin and non‐kin, which was conceptually similar to models of inclusive fitness (Hamilton, 1964a, 1964b). However, models of kin selection in the evolution of altruism focus on the actor of the altruistic behavior, whereas our model focuses on the receivers of the cues rather than the senders. Although the evolution of the receiver's sensitivity can be driven by kin selection, the results are not comparable to those of the evolution of cooperation due to the difference in the model structure. Typically, the receiver's response does not benefit the sender of cues, contrasting with cooperation that provides advantages for the partner. The study proposed a new view of kin selection and successfully revealed a potential dilemma for the cue receivers between positive effects due to induced resistance and negative effects via competition with kin. The result of our conceptual model highlights the importance of competitive regimes in the evolution of signaling among kin, providing a significant factor that is required in order to understand kin communication.

In the present analysis, our focus lies on examining the response of receivers in a scenario where infested plants emit warning cues, while uninfested plants do not. This assumption relaxes the requirements for the evolution of a warning system among kin, as it assumes the presence of pre‐existing kinship information within the warning cues. However, even under these favorable conditions, we have observed that the receiver's response to kin‐related information may not be consistent. This highlights the intricate nature of the evolution of kin recognition, which cannot be reduced to a simplistic explanation. While our investigation identifies the sufficient conditions for the evolution of a warning system accompanied by kin recognition, incorporating the evolution of kinship information within the warning cues into the model holds promise for future studies.

## AUTHOR CONTRIBUTIONS

**Atsushi Yamauchi:** Conceptualization (lead); formal analysis (lead); funding acquisition (lead); investigation (lead); methodology (lead); project administration (lead); software (lead); validation (lead); visualization (lead); writing – original draft (lead). **Junji Takabayashi:** Conceptualization (equal); validation (equal); writing – review and editing (equal). **Kaori Shiojiri:** Conceptualization (equal); validation (equal); writing – review and editing (equal). **Richard Karban:** Conceptualization (equal); validation (equal); writing – review and editing (lead).

## FUNDING INFORMATION

JSPS KAKENHI Grant Number 19K06851 for AY. JSPS KAKENHI Grant Number 22H00425 for JT.

## CONFLICT OF INTEREST STATEMENT

We know of no conflicts of interest associated with this publication, and there has been no significant financial support for this work that could have influenced its outcome. As the corresponding author, I confirm that the manuscript has been read and approved for submission by all the named authors.

## Data Availability

Mathematica notebooks are uploaded to Zenodo. https://doi.org/10.5281/zenodo.10604574.
